# A Joint Constraint Incentive Mechanism Algorithm Utilizing Coverage and Reputation for Mobile Crowdsensing

**DOI:** 10.3390/s20164478

**Published:** 2020-08-11

**Authors:** Jing Zhang, Xiaoxiao Yang, Xin Feng, Hongwei Yang, An Ren

**Affiliations:** 1 College of Computer Science and Technology, Chang Chun University of Science and Technology, Changchun 130022, China; zhang_jing@cust.edu.cn (J.Z.); 2018100591@mails.cust.edu.cn (X.Y.); hongweiyang@cust.edu.cn (H.Y.); 2 Petrochina Research Institute of Petroleum Exploration and Development, Beijing 100083, China; renan@petrochina.com.cn

**Keywords:** mobile crowdsensing, coverage, historical reputation, Stackelberg game theory, incentive mechanism

## Abstract

Selection of the optimal users to maximize the quality of the collected sensing data within a certain budget range is a crucial issue that affects the effectiveness of mobile crowdsensing (MCS). The coverage of mobile users (MUs) in a target area is relevant to the accuracy of sensing data. Furthermore, the historical reputation of MUs can reflect their previous behavior. Therefore, this study proposes a coverage and reputation joint constraint incentive mechanism algorithm (CRJC-IMA) based on Stackelberg game theory for MCS. First, the location information and the historical reputation of mobile users are used to select the optimal users, and the information quality requirement will be satisfied consequently. Second, a two-stage Stackelberg game is applied to analyze the sensing level of the mobile users and obtain the optimal incentive mechanism of the server center (SC). The existence of the Nash equilibrium is analyzed and verified on the basis of the optimal response strategy of mobile users. In addition, mobile users will adjust the priority of the tasks in time series to enable the total utility of all their tasks to reach a maximum. Finally, the EM algorithm is used to evaluate the data quality of the task, and the historical reputation of each user will be updated accordingly. Simulation experiments show that the coverage of the CRJC-IMA is higher than that of the CTSIA. The utility of mobile users and SC is higher than that in STD algorithms. Furthermore, the utility of mobile users with the adjusted task priority is greater than that without a priority order.

## 1. Introduction

Mobile crowdsensing (MCS), as a new and rapidly emerging information collection paradigm, has aroused extensive concerns for solving complex sensing problems [1,2]. Complex sensors (e.g., cameras, GPS, and microphones) in mobile smart devices provide superior tools for acquiring sensing data in MCS network systems. At present, MCS has been widely used in water pollution [3], environmental monitoring [4], health services [5], intelligent transportation [6], and other fields. In MCS, mobile users (MUs) consume limited resources to complete sensing tasks in their spare time, and they may face potential threats of privacy disclosure (e.g., geographic location). Therefore, designing a reasonable incentive mechanism to encourage more MUs to participate in the sensing task and maximize the quality of the sensing data in MCS is a hot issue.

To collect high-quality sensing data, scholars have proposed numerous incentive mechanisms for MCS network systems [7,8,9,10]. The incentive mechanism is the core of the MCS network [11]. Existing incentive mechanisms can be roughly divided into monetary [12,13,14,15,16,17,18,19] and non-monetary incentive mechanisms [20,21]. Compared with the non-monetary incentive mechanism, the monetary one is a more flexible reward method. The monetary incentive mechanism often works better than the non-monetary incentive mechanism in [22].

Game theory can provide an excellent mathematical model to solve the balance problem of utility between the SC and the MUs in the monetary incentive mechanism. Different game models are used to simulate and solve various problems in diverse scenarios. A RADP incentive mechanism based on the reverse auction, in which users can sell sensing data to a service provider, is designed [12]. VCG auction policy is proposed for online sensor selection [13], and the sensor selection problem is considered a general time-dependent and location-aware participatory sensing system. Two optimization models are proposed to solve the problem of heterogeneous costs across the sensing area in [14]. The platform-centric model is constructed as a Stackelberg game, which executes only one sensing task in [15]. The incentive mechanism based on the Stackelberg game is applied to spectrum sensing in [16]. The incentive mechanism based on the Stackelberg game is also used to build a model of the novel rumor control framework in the mobile social network in [17]. A new incentive mechanism of QUOIN based on the Stackelberg game is investigated to combine the MCS network and big data in [18]. The authors of [19] proposed discriminatory and uniform incentive mechanisms, which considered the social network underlying the mobile social domain.

However, the coverage of MUs in the target area is always ignored, especially being absent in the above papers. The coverage rate of the collected sensing data is related to the accuracy of sensing data in the target area. The entire sensing region is divided into several equal-sized blocks, which are mentioned in [23,24], and sensing data are collected from different blocks and are profiled for the entire region accurately [25]. Optimal MUs are selected using the coverage as an indicator in the literature [26,27]. Nevertheless, the quality of the sensing data uploaded cannot be guaranteed during the task. The historical reputation of an MU reflects its previous behavior [28], which is used as a parameter for selecting the optimal MUs to minimize the threat from dishonest users. Therefore, this study aims to find optimal MUs whose data can meet the information quality requirements of the SC and task publisher (TP) better. The optimal MUs are selected on the basis of the historical reputation of MUs. Thus, the unbelievable behavior of MUs is restricted to protect the benefits of the SC and task publisher (TP) [28].

This study aims to design an incentive mechanism for maximizing the utility of MUs and the SC, and the MUs with a high reputation will be encouraged to participate and collect high-quality sensing data in the sensing task. The primary contributions of this study are summarized as follows:

- An optimal MU selecting algorithm (OMUS) is proposed to select the optimal MUs according to the location information and historical reputation of MUs. Thus, the collected sensing data will be more accurate and credible;
- A two-stage Stackelberg game model is proposed to solve the balance problem between the lowest rewards of the SC and the optimal strategy of the MUs in the MCS system, and the existence of the Nash equilibrium is proven in the Stackelberg game;
- A task priority time series method is proposed to maximize the total utility of the MUs’ tasks;
- A reputation update and reward allocation method for the MUs is proposed. After the MUs upload the sensing data, the EM algorithm is used to evaluate the quality of sensing data, and SC evaluates the reputation of MUs according to the quality of sensing data and updates the historical reputation of each MU. Then, the reward is allocated to MUs who have completed the tasks according to the selected optimal strategy.

## 2. System Model and Game Formulation

**Figure 1:**
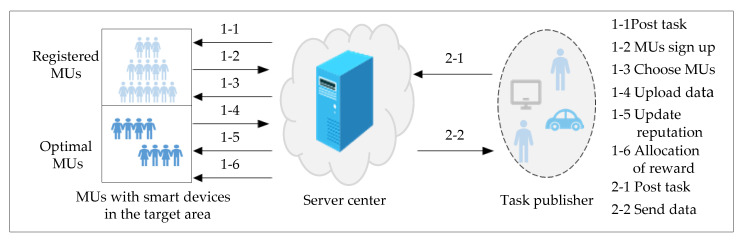
System model of crowdsensing.

The MCS network system includes the TP, the SC, and the MUs in this paper. MUs are equipped with various mobile smart devices (e.g., smartphones, tablets). This work describes a procedure in which MUs receive and accomplish the sensing task when the TP publishes a task to the SC. In Figure 1, the SC will broadcast the task to MUs located in the target area when the TP uploads the sensing task to the SC. The set of MUs that have signed up for the sensing task is *U* = {*u*_1_, *u*_2_, …, *u_n_*}, and the SC selects the optimal MUs to participate in the task. MUs perform the sensing task and upload the sensing data to the SC. Finally, the EM algorithm is used to evaluate the quality of the sensing data, and the SC updates the reputation of MUs. Meanwhile, the SC allocates reward to MUs for the task, and the SC sends the sensing data to the TP.

The detailed process is presented as follows.

(1) TP publishes a sensing task and the target area to the SC;
(2) If the MUs with a mobile smart device sensor are interested in the sensing task, then they will sign up to participate in the sensing task. The MU set is *U* = {*u*_1_, *u*_2_, …, *u_n_*};
(3) The SC uses the OMUS algorithm to select the optimal users *W* = {*w*_1_, *w*_2_, …, *w_m_*} (*m* ≤ *n*);
(4) The SC and the selected MUs choose their optimal strategies by using the coverage and reputation joint constraint incentive mechanism algorithm (CRJC-IMA). When the SC determines the total reward *R,* MUs choose the optimal bandwidth strategy to maximize the utility of the SC and MUs;
(5) Each MU sorts the tasks in time series according to the allocated reward to maximize the total utility of the MUs;
(6) MUs upload sensing data to the SC, and MUs receive the reward allocated by the SC;
(7) The SC evaluates the quality of the sensing data, and the SC updates the reputation of MUs.

The main parameter definitions in this work are shown in Table 1.

The TP publishes a sensing task and allocates the total reward *R* to the MUs who perform the sensing task, where *R* > 0. When the MUs participate in the task, the SC will acquire the payoff, which can be expressed as a function ρ(·). The payoff function ρ(·) increases with the MUs’ sensing level and decreases with the increase in the sensing rate.

(1) $$\rho \left(Sc\right)=\lambda_{1}\ln\left(1+\overset{m}{\underset{i=1}{\sum }}\ln\left(1+B_{i}\right)\right)+\lambda_{2}\ln\;\left(1+\overset{m}{\underset{i=1}{\sum }}\ln\left(1+E_{i}\right)\right)$$

where $\lambda_{1}$ and $\lambda_{2}$ are system parameters; and $B_{i}$ and $E_{i}$ are the bandwidth strategy selected by MU*_i_* and the energy consumed by MU*_i_* for transmitting data, respectively. The utility of the SC can be expressed as

(2) $$u_{0}^{\prime }=\rho \left(Sc\right)-R$$

To maximize the utility of MUs, they will not participate in sensing tasks unless incentives are sufficient. The payoff and cost functions of MUs in this work are determined by the energy consumption and bandwidth used by MUs to perform the task. The payoff function *f_i_* and cost function $g_{i}$ of each MU can be defined as

(3) $$f_{i}=\left(\frac{B_{i}}{\sum_{i=1}^{m}B_{i}}+\frac{E_{i}}{\sum_{i=1}^{m}E_{i}}\right)*\frac{R}{2}$$

(4) $$g_{i}=\alpha_{i}E_{i}+\beta_{i}B_{i}$$

The utility of MU*_i_* can be defined as

(5) $$u_{i}=f_{i}-g_{i}$$

The utility of each MU is composed of payoff and cost functions. The payoff function *f_i_* is formed by the bandwidth and energy consumed by MU*_i_*. Bandwidth $B_{i}$ is the strategy selected by MU*_i_*. Energy $E_{i}$ is determined by the distance from the SC to MU*_i_* when the sensing data are transmitted between them. The function $g_{i}$ is determined by the energy and bandwidth cost. $\alpha_{i}>0$ and $\beta_{i}>0$ are the unit cost of the energy and bandwidth of MU*_i_*, respectively.

The energy consumption of MU*_i_* mainly includes the energy consumption of sending and receiving data during the sensing task, and other energy consumption used by MUs can be ignored [29]. Equation (6) represents the energy consumption of transmitting and receiving data.

(6) $$E\left(k,d\right)=\{\begin{array}{c} k*E_{elect}+k*\varepsilon_{fs}*d^{2},d\leq d_{0}\\ k*E_{elect}+k*\varepsilon_{amp}*d^{4},d>d_{0} \end{array}$$

where *E_elect_* represents the energy consumption of sending and receiving *k* bit data, *d* is the transmission distance between the SC and each MU, *d*_0_ is the distance threshold equal to 87 m, and *_fs_* and *_amp_* are the amplifier power consumption of the free space model and multipath attenuation model, respectively. When the distance between MU*_i_* and SC is less than *d*_0_, the free space model is adopted, and the transmission power is attenuated to *d*^2^. Otherwise, the multipath attenuation model is adopted, and the transmission power is set as *d*^4^.

In this work, the number of sensing tasks performed by each MU is greater than or equal to 1. When the published tasks and determined rewards by the SC vary, the MU acquires payoff, and utility is distinct after performing the tasks. Suppose *h* tasks are needed to be completed by MU*_i_*, and a device can only perform one task during the execution of the task. The required time for each task is {*t_i_*_1_, *t_i_*_2_, …, *t_ih_*}. The corresponding utility of *h_i_* tasks are {*u_i_*_1_, *u_i_*_2_, …, *u_ih_*}. Therefore, the priority of *h* tasks performed by MU*_i_* is {*p_i_*_1_, *p_i_*_2_, …, *p_ih_*}. Then, the total required time that MU*_i_* executes task *l* is $t_{il}^{’}=\overset{h=l-1}{\underset{h=1}{\sum }}t_{ih}+t_{l}$. To maximize the total utility of MU*_i_*, the priority of task *l* performed by MU*_i_* is

(7) $$p_{il}\;s.t.\;\max\overset{l=h}{\underset{l=1}{\sum }}u_{il}$$

The utility of MU*_i_* with task priority considered can be defined as

(8) $$u_{il}^{0}=u_{il}-\gamma_{i}t_{il}^{\prime }$$

where $\gamma_{i}$ is the unit time MU*_i_* costs to accomplish the sensing tasks. Each MU*_i_* spends different times on distinct tasks. Thus, various utilities for each MU*_i_* in different tasks can be defined as Equation (8). The tasks performed by MU*_i_* will be sorted in descending order according to the utility of the tasks. Therefore, the task with the maximum utility will be executed first.

## 3. CRJC-IMA

In this work, the whole CRJC-IMA consists of four phases. The first phase selects the optimal MUs. In the second phase, the follower and leader games are applied to analyze the sensing level of the MUs and the optimal incentive mechanism of the SC, respectively. The third phase includes the quality evaluation process in which the MUs upload sensing data and the historical reputation value update process. The fourth stage performs the incentive allocation process. The game process will be described in the next section.

### 3.1. OMUS

The selection of optimal MUs for completing the task is a challenge for the MCS network system. The MUs with a higher reputation and wider coverage are superior to collect high-quality sensing data for the TP than other MUs. The number of selected *m* MUs is related to the total reward *R* of the TP and the cost of registered MUs. The registered MUs are MUs signed up to participate in the sensing task. The number of users *m* can be determined as follows.

(9) $$m=\frac{R}{2\left(\mu_{1}\alpha_{sum}\bar{E}+\mu_{2}\beta_{sum}\bar{B}\right)}$$

where α*_sum_* is the energy unit cost sum of the registered Mus, β*_sum_* is the bandwidth unit cost sum of the registered Mus, $\bar{E}$ is the mean value of the maximum and minimum consumption energy of registered MUs in the process of data transmission, $\bar{B}$ is the average of the maximum and minimum bandwidth selected by registered Mus, and $\mu_{1}$ and $\mu_{2}$ are system parameters.

(10) $$\alpha_{sum}=\overset{n}{\underset{i=1}{\sum }}\alpha_{i},\beta_{sum}=\overset{n}{\underset{i=1}{\sum }}\beta_{i},\bar{E}=\frac{E_{max}+E_{min}}{2},\bar{B}=\frac{B_{max}+B_{min}}{2}$$

**Figure 2:**
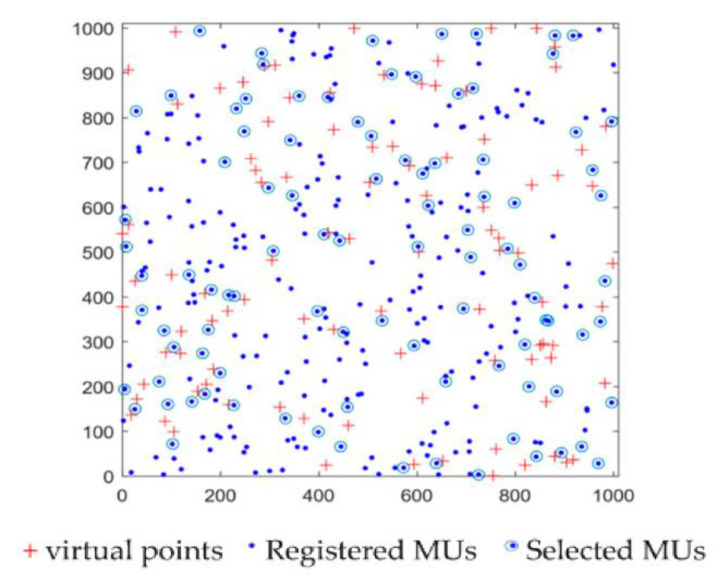
Virtual points and selected optimal mobile users (MUs).

After the SC publishes a sensing task, the MUs in set *U* = {*u*_1_, *u*_2_, ..., *u_n_*} sign up to complete the published task in the MCS network. First, the SC selects the optimal virtual points in the target area. Then, the SC determines the optimal MUs based on geographic location information and the historical reputation of the MUs according to virtual points. The selected virtual points and MUs are shown in Figure 2.

#### 3.1.1. Virtual Point Selection (VPS)

The PSO [30] has the characteristics of fast search speed and high efficiency. Therefore, the PSO is applied to select *m* virtual points with maximum coverage in the target area randomly.

**Definition** **1.** *Area coverage. It is the ratio of the sensing area* s *to the target area*  S *perceived by MUs.*

The fitness of PSO is the coverage rate $f=s_{1}/S$. *s*_1_ is the area covered by the virtual points, and S is the target area.

The specific process of PSO is presented as follows.

Step 1: Parameter setting. Initializing the speed and position of the *m* virtual points randomly in the target area;
Step 2: Calculating the coverage of *m* virtual points and finding the individual extremum and group extremum. The individual extremum is the coverage rate of the virtual point’s optimal position, and the group extremum is the optimal position of the virtual point corresponding to the maximum coverage among the individual extremes of all virtual points;
Step 3: Updating the speed and position of each virtual point in the virtual points set;
Step 4: Calculating the coverage of the *m* virtual points;
Step 5: Updating the individual extremum and group extremum of the *m* virtual points;
Step 6: If the maximum number of iterations has been reached, then the global optimal position will be determined, otherwise skip to the second step.

According to the above steps, *m* virtual points are randomly selected.

#### 3.1.2. MU Selection Process

Through the above proposed VPS algorithm, *m* virtual points are selected in the target area of the MCS. Then, *m* MUs are selected to perform the task from the *n* MUs signed up for the task according to *m* virtual points. The objective function between the virtual point *j* and each MU*_i_* can be defined as

(11) $$f_{ij}=a*Cre_{i0}+b*\left(1/d_{ij}\right)$$

where *a* and *b* are the weighting factors and a+b=1, *Cre*_i0_ is the historical reputation of MU*_i_*, and *d_ij_* is the Euclidean distance between MU*_i_* and virtual point j. The SC selects an optimal MU with the largest objective function value according to a virtual point. Thus, the SC will select *m* optimal MUs according to *m* virtual points to perform the sensing task. Therefore, the quality of the collected sensing data can be guaranteed. If a new MU participates in the sensing task, then their reputation will set the highest reputation, which is set as 5 in this work. Thus, the new MU with the highest reputation can be selected preferentially to perform the sensing task, and its reputation value will be accumulated when it participates in other sensing tasks in the future.

### 3.2. Update Reputation of MUs

Reputation is an important index to select optimal MUs in the CRJC-IMA. MUs with high reputation will be selected preferentially by the SC to collect sensing data. In this section, the EM algorithm [31] is applied to evaluate the quality of sensing data in a task, and then the SC updates the historical reputation of each MU according to the completion quality of the task.

#### 3.2.1. Quality Evaluation

The quality of the MUs’ sensing data reflects the quality of tasks completed by MUs. For example, the MUs collect urban noise sensing data [32]. Each MU *w_i_* estimates a quality evaluation matrix $e^{w_{i}}$, which is a m×m matrix with elements $e_{rs}^{w_{i}}\in \left[0,1\right],\;r=1,2,\ldots ,m;s=1,2,\ldots ,m$, and the calculation of the quality evaluation matrix $e^{w_{i}}$ will be given in detail in the following specific steps. The quality evaluation matrix is mapped to the quality of the sensing data by the function $q_{i}=g\left(e^{w_{i}}\right)$. Therefore, the spans of the sensing data are divided into {*d*_1_, *d*_2_, …, *d*_*m*_} intervals, which represent the sensing levels of the collected data. The EM algorithm is applied to estimate the quality evaluation matrix $e^{w_{i}}$ of each MU. The converged estimation of an MU’s evaluation matrix indicates the quality of the sensing data, whereas the noise interval distribution suggests the urban noise pollution level. The specific steps are as follows:

Step 1. For each task, t∈T, the index function $I\left(d_{t}^{k}=d_{j}\right)=1$ when the MU’s sensing data $d_{t}^{k}$ fall into the real interval $d_{j}$, and the probability distribution of the real noise interval are initialized as

(12) $$p_{j}^{t}=p\left(d_{t}^{0}=d_{j}\right)=\frac{\sum_{w_{i}\in W_{l}}I\left(d_{t}^{k}=d_{j}\right)}{\left|W_{l}\right|}$$

Step 2. Estimate the likelihood function of the perception probability matrix, and ${\hat{e}}_{rg}^{w_{i}}$ represents the value after t iterations.

(13) $${\hat{e}}_{rg}^{w_{i}}=\frac{\sum_{t\in T^{w_{i}}}p_{r}^{w_{i}}I\left(d_{t}^{w_{i}}=d_{g}\right)}{\sum_{t\in T^{w_{i}}}p_{r}^{w_{i}}},g=1,2,\ldots ,m$$

The true noise interval distribution is estimated as

(14) $${\hat{\pi }}_{r}=\frac{\sum p_{r}^{t}}{\left|T\right|},r=1,2,\ldots ,m$$

Step 3. Estimate the real noise interval. Given the sensing data *G*, the quality evaluation matrix ***E***, and noise interval distribution Π, Bayesian inference is used to estimate the true noise interval *P*. Calculate the true noise interval distribution according to the following formula.

(15) $$P_{r}^{t}=\frac{\pi_{r}\prod_{w_{i}\in W_{t}}\prod_{g}\left(e_{rg}^{k}\right)I\left(d_{t}^{k}=d_{s}\right)}{\sum_{q}\pi_{q}\prod_{w_{i}\in W_{t}}\prod_{g}\left(e_{qg}^{k}\right)I\left(d_{t}^{k}=d_{s}\right)},r=1,2,\ldots ,m$$

Step 4. Convergence. Steps 2–3 are iterated until the two estimates converge (i.e., $\left|{\hat{E}}^{t+1}-{\hat{E}}^{t}\right|<\varepsilon$, $\left|{\hat{P}}^{t+1}-{\hat{P}}^{t}\right|<\eta$, ε>0, η>0). According to the estimation of the quality evaluation matrix $e^{w_{i}}$, a mapping function g(·) can be used to obtain the quality of *w_i_*’s sensing data. Thus, the quality of *w_i_*’s sensing data is $q_{i}$, that is

(16) $$q_{i}=g\left(e^{w_{i}}\right)=\underset{r}{\sum }e_{rr}^{w_{i}}/m$$

#### 3.2.2. Reputation Updated

Through the above quality evaluation of the sensing data process, the sensing data quality of MU*_i_* is $q_{i}$. Then, the reputation value of MU*_i_* will perform the normalization process and be converted to [0,5], that is,

(17) $$Cre_{i}=5*q_{i}/q_{max}$$

where $q_{max}$ is the highest data quality value of an MU who participates in the task. The SC updates the historical reputation of each MU after the task reputation is estimated, that is,

(18) $$Cre_{i}^{\prime }=\left(o*Cre_{i0}+Cre_{i}\right)/\left(o+1\right)$$

where o is the number of historical tasks in which MU*_i_* participated, and $Cre_{i0}$ is the historical reputation value of MU*_i_*.

### 3.3. Incentive Allocation

When the SC determines the total reward *R*, MUs select the optimal bandwidth strategy to perform the task according to the incentive mechanism algorithm based on the Stackelberg game. After MUs upload the sensing data, the SC will allocate the reward to MUs based on the optimal strategy selected by the MUs. Therefore, the final reward for each MU*_i_* is

(19) $$reward_{i}=f_{i}=\left(\frac{B_{i}^{*}}{\sum_{i=1}^{m}B_{i}^{*}}+\frac{E_{i}}{\sum_{i=1}^{m\;}E_{i}}\right)*\frac{R}{2},B_{i}^{*}\neq 0$$

If the MUs participate in the sensing task, then the SC allocates the reward to each MU according to the payoff function. When MU*_i_* selects the optimal bandwidth $B_{i}^{*}=0$, MU*_i_* does not participate in completing the task. Thus, $reward_{i}=f_{i}=0$.

## 4. Designing the Incentive Mechanism Based on Stackelberg Game

This study aims to design an incentive mechanism for maximizing the utility of MUs and SCs, where the MUs with a high reputation will be encouraged to complete the task. Thus, the relationship between the SC and MUs is modeled as a Stackelberg game [33]. The SC is the leader, and the SC will provide the total reward *R* for the task. MUs are the followers, and all MUs select their optimal bandwidth to maximize their utility according to the SC strategy. Then, the SC will adjust its strategy accordingly. The selected optimal bandwidth by MUs in the follower game can be considered a non-cooperative game [15], which is named as the optimal bandwidth determination (OBD) game. However, the sensing task received by MUs is greater than or equal to one. Therefore, according to the utility of the sensing task, MUs should determine the priority of tasks to maximize the total utility.

### 4.1. Follower Game

The SC allocates rewards to each MU based on its energy consumption and bandwidth strategy, and MUs’ energy and bandwidth payoffs account for half of the total reward given by the SC. Once MUs participate in the sensing task, half of the reward *R* will be allocated to MUs according to the energy consumed by the MUs, and the more energy consumed by the MUs, the more reward MUs will obtain. The remaining reward can get more payoff by adjusting the bandwidth. All of the MUs participating in the sensing task are *W*={*w*_1_,*w*_2_,…,*w_m_*}, and the bandwidth strategy set of MUs is *B*=(*B*_1_,*B*_2_,…,*B_m_*), and *B_-i_*=(*B*_1_,*B*_2_,…,*B_i_*_-1_,*B_i_*_+1_,…, *B_m_*) represents the strategy excluding MU*_i_*. Therefore, *B* can be expressed as *B*=(*B_i_, B_-i_*).

(20) $$u_{i}=\left(\frac{B_{i}}{B_{i}+\sum_{j=1,j\neq i}^{m}B_{j}}+\frac{E_{i}}{\sum_{i=1}^{m}E_{i}}\right)*\frac{R}{2}-\alpha_{i}E_{i}-\beta_{i}B_{i}$$

MU*_i_* will choose the optimal bandwidth strategy to maximize the utility of the task and then choose the priority of the task to maximize the total utility of all tasks. The number of each MU*_i_*’s tasks is greater than or equal to one, and the unit time cost of MU_i_ is a determined value. Therefore, the utility function of MU*_i_* is

(21) $$u_{il}^{0}=\left(\frac{B_{i}}{B_{i}+\sum_{j=1,j\neq i}^{m}B_{j}}+\frac{E_{i}}{\sum_{i=1}^{m}E_{i}}\right)*\frac{R}{2}-\alpha_{i}E_{i}-\beta_{i}B_{i}-\gamma_{i}t_{il}^{’}$$

Assume that MU*_i_* receives *l*-1 tasks, the published task by the TP is *l*, which forms the set *T_i_*= {*T*_1_, *T*_2_, …, *T_l_*} with other *l*-1 tasks. The corresponding priority set of each task is *P*= {*p_i_*_1_, *p_i_*_2_, …, *p_il_*}. MU*_i_* selects the priority of the task to maximize the total utility of all received tasks.

#### 4.1.1. Related Definitions

**Definition** **2.** *The Nash equilibrium. A set profile*$B^{*}=\left(B_{i}^{*},B_{-i}^{*}\right)$*is a Nash equilibrium of the OBD game if each MU_i_ satisfies*$u_{i}\left(B_{i}^{*},B_{-i}^{*}\right)\geq u_{i}\left(B_{i},B_{-i}\right)$*, for any*$B_{i}\geq 0$*, where*$u_{i}$ is defined in (20).

**Definition** **3.** *Optimal response strategy. Given*$B_{-i}$*, a strategy is MU_i_’s optimal response strategy, denoted by*$\rho \left(B_{-i}\right)$*, if it is maximized*$u_{i}\left(B_{i},B_{-i}\right)$*over all*$B_{i}\geq 0$.

*The Nash equilibrium ensures the stability of the OBD game algorithm, and each MU chooses the optimal bandwidth strategy according to other MUs.*

#### 4.1.2. Proofs for Properties

**Property 1**: Follower game of the OBD has a unique Nash equilibrium given a total reward *R* published by the SC.

**Proof.**  To certify the optimal bandwidth strategy of MU*_i_*, the utility $u_{i}$ of MU*_i_* is calculated for the first and second derivatives with respect to its bandwidth strategy *B_i_*, which can be obtained by Equation (20).

(22) $$\frac{\partial u_{i}}{\partial B_{i}}=\frac{-B_{i}R}{2{\left(\sum_{i=1}^{m}B_{i}\right)}^{2}}+\frac{R}{2\sum_{i=1}^{m}B_{i}}-\beta_{i}$$

(23) $$\frac{\partial^{2}u_{i}}{\partial^{2}B_{i}}=\frac{-R\left(\sum_{i=1}^{m}B_{i}-B_{i}\right)}{{\left(\sum_{i=1}^{m}B_{i}\right)}^{3}}<0$$

The utility function in Equation (23) is concave with respect to the bandwidth strategy of MUs. Therefore, the optimal strategy is unique when the total reward is fixed.

By setting the first derivative to zero

(24) $$\frac{-B_{i}R}{2{\left(\sum_{i=1}^{m}B_{i}\right)}^{2}}+\frac{R}{2\sum_{i=1}^{m}B_{i}}-\beta_{i}=0$$

Solved by Equation (24)

(25) $$\rho \left(B_{i}\right)=B_{i}^{*}=\sqrt{R\underset{j\in W\backslash \left\{i\right\}}{\sum }B_{j}/2\beta_{i}}-\underset{j\in W\backslash \left\{i\right\}}{\sum }B_{j}$$

When $B_{i}^{*}$ is positive in Equation (25), this bandwidth is the optimal strategy for MU*_i_*. If $B_{i}^{*}$ is negative, then MU*_i_* will not participate in the sensing task and $B_{i}^{*}=0$. □

### 4.2. Leader Game

All participating MUs have a unique Stackelberg equilibrium bandwidth strategy when the SC gives the total reward *R* in the above section. In the leader game, the SC can maximize its utility by adjusting *R*.

**Property 2:** A unique Stackelberg equilibrium (*R^∗^*, B^*^) exists in the leader game.

**Proof.**  From Equation (24), we can obtain

(26) $$\frac{-B_{i}R}{2{\left(\sum_{i=1}^{m}B_{i}\right)}^{2}}+\frac{R}{2\sum_{i=1}^{m}B_{i}}-\beta_{i}=0$$

We sum all participating MUs in *W* for Equation (26) and obtain the following formula:

(27) $$\left(m-1\right)R/2=\underset{i\in W}{\sum }B_{i}\cdot \overset{m}{\underset{i=1}{\sum }}\beta_{i}$$

(28) $$\underset{i\in W}{\sum }B_{i}=\left(m-1\right)R/2\overset{m}{\underset{i=1}{\sum }}\beta_{i}$$

Substituting Equation (28) into Equation (26) yields

(29) $$B_{i}=\left[\left(m-1\right)R/2\overset{m}{\underset{i=1}{\sum }}\beta_{i}\right]\cdot \left[1-\left(m-1\right)\beta_{i}/\overset{m}{\underset{i=1}{\sum }}\beta_{i}\right]$$

Substituting Equation (29) into the SC utility function yields

(30) $$u_{0}^{\prime }=\lambda_{1}\ln\left\{1+\overset{m}{\underset{i=1}{\sum }}\ln\left[1+\left[\left(m-1\right)R/2\overset{m}{\underset{i=1}{\sum }}\beta_{i}\right]\cdot \left[1-\left(m-1\right)\beta_{i}/\overset{m}{\underset{i=1}{\sum }}\beta_{i}\right]\right]\right\}+\lambda_{2}h_{0}^{\prime }\left(E_{i}\right)-R$$

(31) $$h_{0}^{\prime }\left(E_{i}\right)=\ln\left(1+\overset{m}{\underset{i=1}{\sum }}\ln\left(1+E_{i}\right)\right)$$

Solving the second derivative of the SC utility function obtains

(32) $$\frac{\partial^{2}u_{0}^{\prime }}{\partial R^{2}}=-\lambda_{1}\left[\overset{m}{\underset{i=1}{\sum }}\left(F_{i}^{2}\cdot K_{i}\right)/{\left(1+F_{i}R\right)}^{2}+{\left[\overset{m}{\underset{i=1}{\sum }}F_{i}/\left(1+F_{i}R\right)\right]}^{2}\right]/K_{i}^{2}<0$$

where

(33) $$K_{i}=1+\overset{m}{\underset{i=1}{\sum }}\ln\left[1+\left[\left(m-1\right)R/2\overset{m}{\underset{i=1}{\sum }}\beta_{i}\right]\cdot \left[1-\left(m-1\right)\beta_{i}/\overset{m}{\underset{i=1}{\sum }}\beta_{i}\right]\right]$$

(34) $$F_{i}=\left[\left(m-1\right)/2\overset{m}{\underset{i=1}{\sum }}\beta_{i}\right]\cdot \left[1-\left(m-1\right)\beta_{i}/\overset{m}{\underset{i=1}{\sum }}\beta_{i}\right]$$

Therefore, the proposed algorithm obtained from Equation (32) is strictly concave, and a unique Stackelberg equilibrium (*R^∗^*, B^*^) exists in the leader game. A unique maximizer *R^∗^* can be computed efficiently using Newton’s method [34]. □

## 5. Simulation Results and Analysis

Simulation experiments are conducted using MATLAB R2016a to evaluate the effectiveness of the CRJC-IMA. A total of 1 TP, 1 SC, and 1000 MUs are distributed randomly in the target area with the range of 1 × 1 km, and each MU’s sensing range is 60 m. The TP can successfully publish tasks to the SC. The parameters and experimental values of this paper are listed in Table 2.

### 5.1. Performance Evaluation of Selecting the Optimal Users

This section evaluates the optimal number of MUs, coverage rate, and reputation in the OMUS. The CRJC-IMA is proposed in the MCS scenario, and the reputation and the coverage of the MUs are important indicators for selecting the optimal MUs. The CTSIA [27] is an incentive mechanism based on reverse auctions under total reward constraints, which have a good coverage ratio in the target area. Therefore, the CRJC-IMA has compared the coverage ratio with the CTSIA under a certain reward.

**Figure 3:**
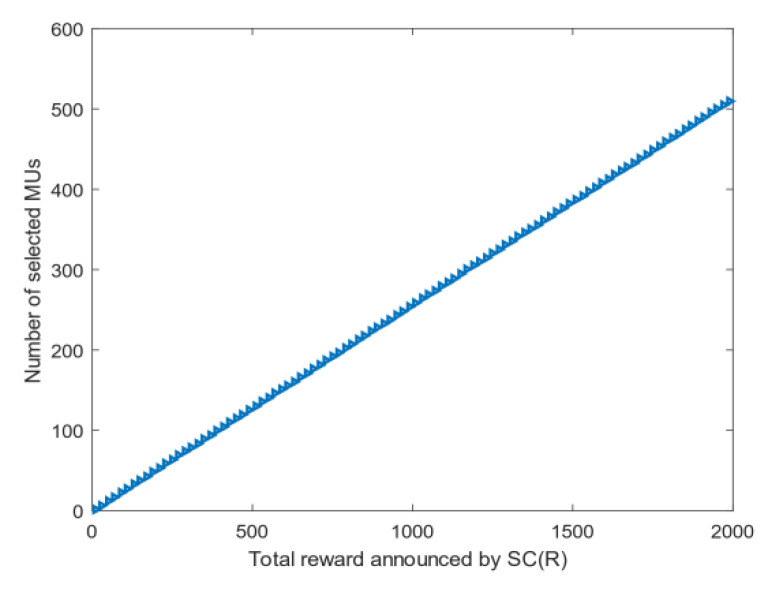
Relationship between the optimal number of MUs and total reward.

**Figure 4:**
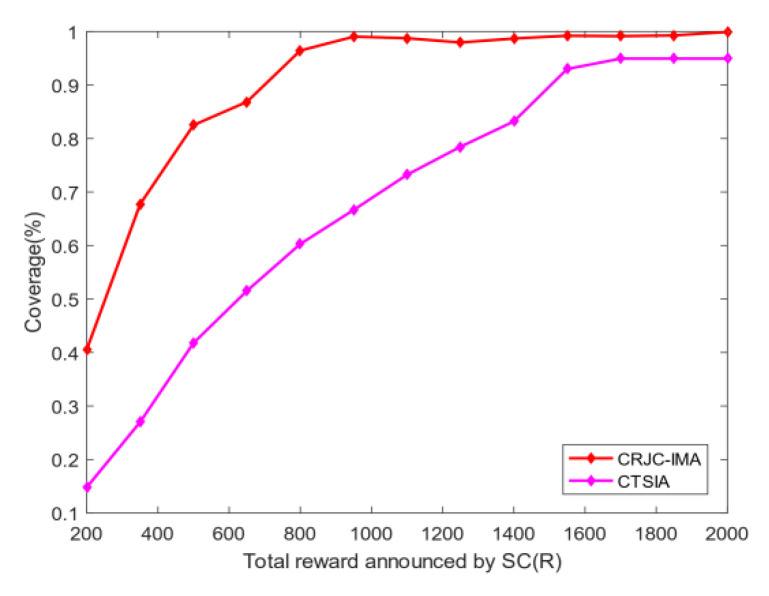
Change of coverage and total reward.

**Figure 5:**
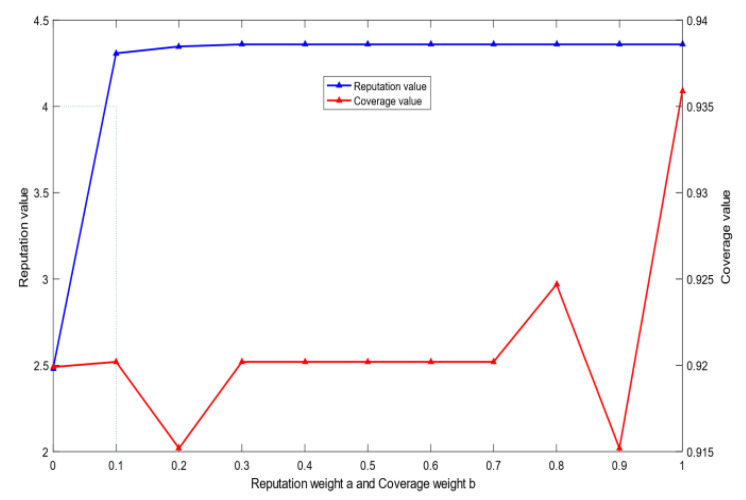
Change reputation and coverage with their weight.

1. The number of optimal users: Figure 3 shows the relationship between the optimal number of MUs *m* and the total rewards *R* given by the SC in the CRJC-IMA. As the rewards increase to 2000, an increasing number of MUs are selected as the collectors to sense the data and the number of selected MUs rises linearly. This phenomenon indicates that the CRJC-IMA performs well relative to the reward.
2. Coverage: Figure 4 shows the relationship between the total reward *R* given by the SC and the coverage rate of the CRJC-IMA and CTSIA algorithms. The coverage rate in the target area is increasing in both algorithms as the reward *R* rises. When the total reward *R* is greater than 800, the coverage of the CRJC-IMA reaches 90%. When *R* is greater than 1600, the coverage rate of both algorithms will not increase significantly, and both reach 90%. However, more reward will increase the cost of the SC and cause excessive data redundancy. The experimental results of the two algorithms show that the coverage rate of the CRJC-IMA is superior to that of the CTSIA.
3. Reputation: Figure 5 shows the influence of different weights on reputation and coverage when selecting the optimal users using Equation (11). The horizontal axis represents a and b, whose domains are between 0 and 1. When the horizontal axis represents the reputation weight a, the vertical axis that corresponds to the left represents the reputation value. The vertical axis that corresponds to the right represents the coverage value when the horizontal axis represents the coverage weight b. The sum of the reputation weight a and coverage weight b is 1. For example, the coverage weight b is 0.9 when the reputation weight a is 0.1. The experimental result shows that the reputation value of the selected MUs is a non-decreasing trend with the increase in reputation weight. When the weight of reputation is greater than 0.1, the average reputation value of the users will reach 4. The coverage of the selected MUs is related to the number of users. Thus, the coverage ratio and weight have a weak coupling relationship.

### 5.2. Performance Evaluation of Incentive Mechanism

**Figure 6:**
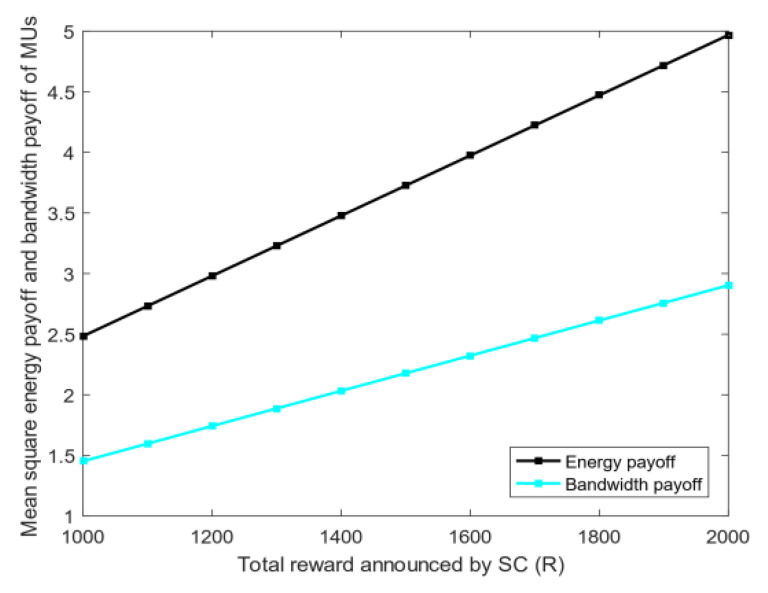
Variation in the mean square of MUs’ energy payoff and bandwidth payoff with *R*.

**Figure 7:**
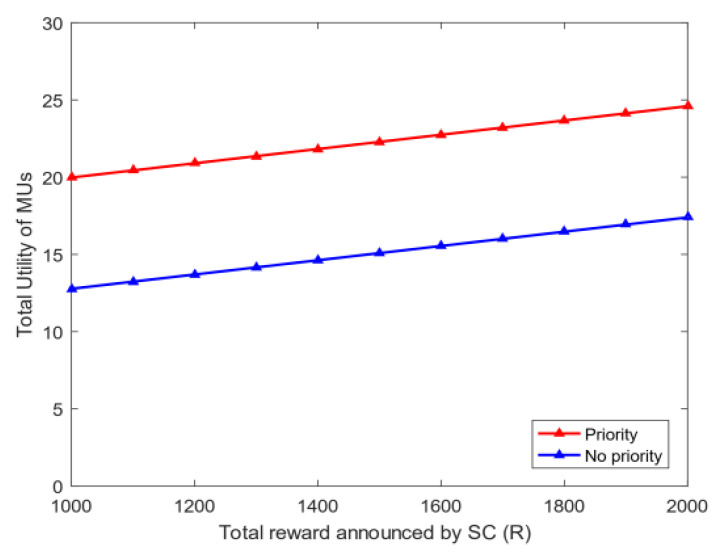
Changes in total utility and *R* of MUs whether choosing priority.

**Figure 8:**
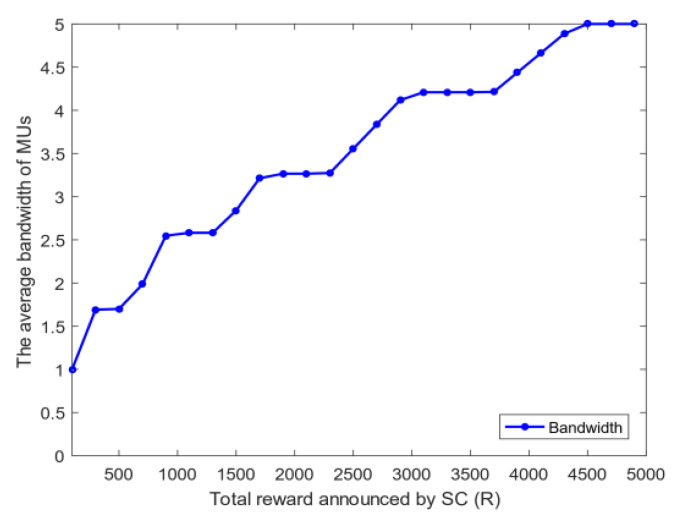
Average bandwidth selected by MUs when *R* changes.

**Figure 9:**
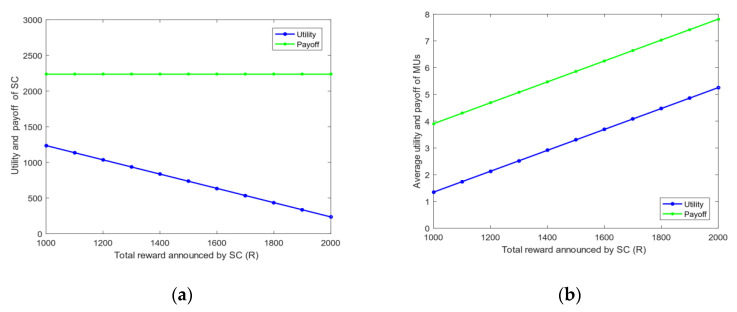
(**a**) Change in server center (SC) utility and payoff with total reward *R*; (**b**) change in average utility and payoff of MUs with total reward *R*.

**Figure 10:**
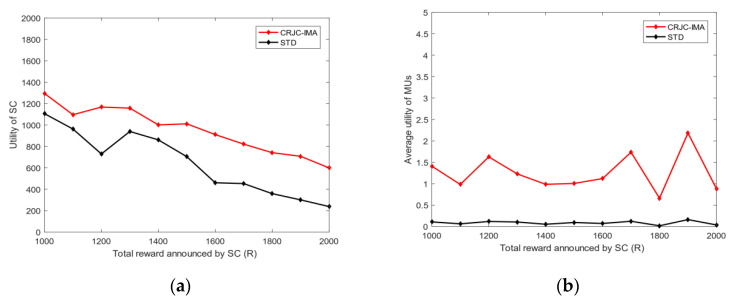
(**a**) Utility of the SC changes with *R*; (**b**) average utility of MUs changes with *R*.

**Figure 11:**
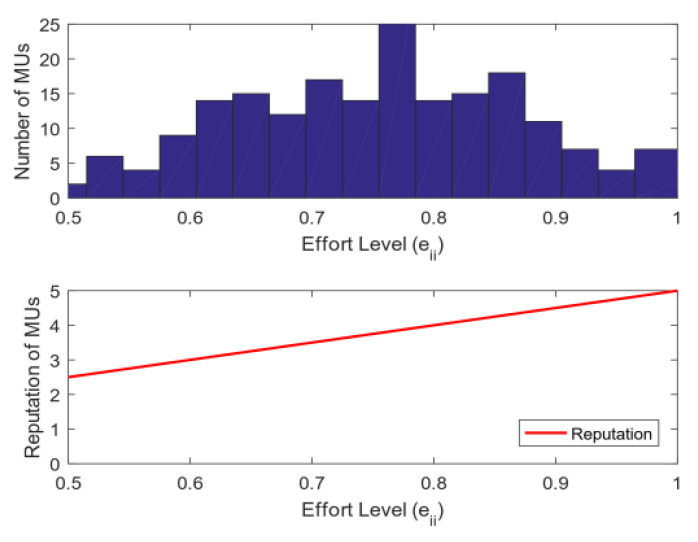
Number and reputation of MUs change with the effort level.

The SC will select optimal MUs in the target area when the SC publishes the total reward *R* to the MUs for completing the task. Figure 5 shows that the weights of the optimal MUs’ reputation and coverage are set as 0.2 and 0.8, respectively. Figure 6, Figure 7, Figure 8, Figure 9, Figure 10 and Figure 11 analyze the MUs’ energy payoff and bandwidth payoff, the utility of MUs selection priority, the bandwidth strategy selected by MUs, the utility and payoff of MUs and the SC, and evaluation of the reputation of MUs, respectively.

4. Energy and bandwidth payoff: Figure 6 analyzes the mean square deviation of the energy payoff and bandwidth payoff obtained by the MUs. The total reward R given by the SC to MUs will be divided into two parts: each MU is rewarded on the basis of the energy consumed and bandwidth used. As shown in Figure 6, the mean square deviation of the energy payoff and bandwidth payoff increases with the total reward R. This phenomenon shows that the gap among MUs’ energy payoff, bandwidth payoff, and the average payoff increases with R. The mean square deviation of the energy payoff is greater than that of the bandwidth payoff when R is fixed because energy is determined by the distance of MUs, and the bandwidth is selected between zero and five. Thus, the stability of each MU’s energy payoff is worse than that of the bandwidth payoff.
5. The utility of priority: Figure 7 shows the relationship between the utility of MUs whether choosing priority and the total reward R of the task. The task has the utility when MUs choose the optimal bandwidth strategy. However, if MUs need to perform other tasks, then the utility of each task will vary because the total rewards of the MU’s every task are diverse. Figure 7 illustrates that as the total reward R of the task increases, the total utility obtained by MUs also rises. After the priority ranking is performed, the total utility obtained by MUs will be greater than that without prioritization. This finding shows that the total utility will increase after each MU selects the priority of the task, and the MU can perform tasks better to avoid time conflicts.
6. Bandwidth strategy: Figure 8 analyzes the relationship between the bandwidth selected by MUs and the total reward R of the task. In Figure 8, the average bandwidth selected by MUs increases with the total reward R. When the total reward is less than 1000, the average bandwidth selected by the MU is less than 2.5. However, MUs choose the average bandwidth value to exceed 4 when the total reward is greater than 3000.
7. Utility and payoff: Figure 9 shows the utility and payoff of MUs in the follower game and the utility and payoff of the SC in the leader game when the MUs have been selected to perform the task. The horizontal axis represents the total reward. In Figure 9a, the utility of the SC is declined when the total reward R increases from 1000 to 2000, and the payoff of the SC remained unchanged. Considering the selected MUs are certain, the utility of the SC will decrease when the total reward paid by the SC increases. In Figure 9b, the average utility and payoff of MUs are growing with the increase in R. Moreover, the total rewards paid by the SC are linearly related to the average utility and payoff of MUs. This indicates that under the condition of a certain number of users, the more rewards the SC has, the less utility the SC has, however, the users will have more utility.
8. Figure 10 shows the utility of the SC and MUs of the CRJC-IMA and STD algorithm [15]. The STD algorithm is a non-cooperative game based on the Stackelberg game, and the total reward and sensing time of a task are used as the parameters of the utility function in the proposed game formula. With the increase in the total reward R paid by the SC to MUs, the utility of the SC in both algorithms declines (Figure 10a). As R grows, the optimal number of MUs increases, and the average utility of the two algorithms will no longer increase significantly (Figure 10b). However, MUs have more average utility when the total reward R and the optimal number of users are constant in the CRJC-IMA. The reason is that the cost is determined by the bandwidth of MUs when performing the task in the CRJC-IMA, and the cost of MUs will be lower than that of the STD algorithm.
9. Reputation evaluation: Figure 11 shows the relationship between the reputation of MUs and the quality evaluation matrix (effort level eii) of the sensing data collected by MU wi. For the quality evaluation matrix eii of the sensing data, the majority of MUs uploaded follow the same normal distribution in [32], where μ = 0.75 and σ = 0.125. Figure 11 shows a linear relationship between the effort level (eii) and the reputation. The MU’s reputation is lower when the MU uploads the sensing data with a small evaluation matrix. However, the MUs’ reputation will be higher if the evaluation matrix of the MUs is larger.

## 6. Conclusions

In this work, an incentive mechanism is proposed on the basis of the Stackelberg game that considers coverage and reputation for MCS. The overall mechanism includes the OMUS algorithm, two-stage Stackelberg game model, task priority time series method, and reputation update and reward allocation method. The accuracy of the collected sensing data has an obvious improvement, because the optimal MUs are selected by the constraint of coverage and reputation. In addition, each user chooses the sensing task priority to ensure that the utility of tasks reaches the maximum. Compared with the CTSIA, the proposed model in this paper has a higher coverage rate. The utility of the SC and MUs in the proposed method is greater than that in the STD algorithm. Meanwhile, MUs have more utility than those without a priority order. However, this article does not consider the user selection task problem when multiple tasks are released. Therefore, an incentive mechanism considering multiple TPs will be investigated in future work. Moreover, we will design an incentive mechanism that combines non-monetary incentives to reduce the total rewards of the SC in the MCS.

## Figures and Tables

**Table 1:** Definitions of notations.

Symbol	Definition	Symbol	Definition
*U* = {*u*_1_, *u*_2_, …, *u_n_*}	Registered users set	*d_ij_*	Distance between MU*_i_* and point *j*
*W* = {*w*_1_, *w*_2_, …, *w_m_*}	Optimal MUs	$Cre_{i0}$	The historical reputation of MU*_i_*
*m*	Choose the number of optimal MUs	*f_ij_*	The objective value of MU*_i_* and point *j*
*R*	Total reward	*u_i_*	MU*_i_* chooses the utility of the task
*r*	Sensing range	$u_{il}^{0}$	Utility after MU*_i_* chooses priority
ρ(·)	Payoff of SC	*h*	Number of the tasks for MU
*B_i_*	Bandwidth strategy MU*_i_* selected	*p_il_*	Priority for MU*_i_* to perform task *l*
*E_i_*	Energy used by MU*_i_*	$t_{il}^{\prime }$	Time for MU*_i_* to perform task *l*
$u_{0}^{\prime }$	Utility of SC	*E_elect_*	Radio electronic energy
$f_{i}$	Payoff of MU*_i_*	*ε_fs_*	Radio amplifier energy
$g_{i}$	Cost of MU*_i_*	*ε_amp_*	Radio amplifier energy
$d_{0}$	Threshold	*k*	Packet size

**Table 2:** The value of the simulation parameters.

Parameter	Value
the target area	1000 m *×* 1000 m
n	1000
$\lambda_{1}$	220
$\lambda_{2}$	300
$B_{i}$	[1,5]
$\alpha_{i}$	[2,10]
$\beta_{i}$	[1,5]/3
$k_{i}$	[1,5]
$\gamma_{i}$	[0.5,1]
r	60 m
